# Supplementary material for: Actin Branching Regulates Cell Spreading and Force on Talin, but not Activation of YAP
Source: bioRxiv. 2025 May 11:2025.05.09.653153. Preprint. [Version 1] doi: 10.1101/2025.05.09.653153 (PMC12248056; doi:10.1101/2025.05.09.653153)
Supplement: 1 [file NIHPP2025.05.09.653153V1-supplement-1.pdf]

bioRxiv preprint doi: https://doi.org/10.1101/2025.05.09.653153; this version posted May 11, 2025. The copyright holder for this preprint (which was not certified by peer review) is the author/funder, who has granted bioRxiv a license to display the preprint in perpetuity. It is made available under aCC-BY 4.0 International license.

Supplemental Figures

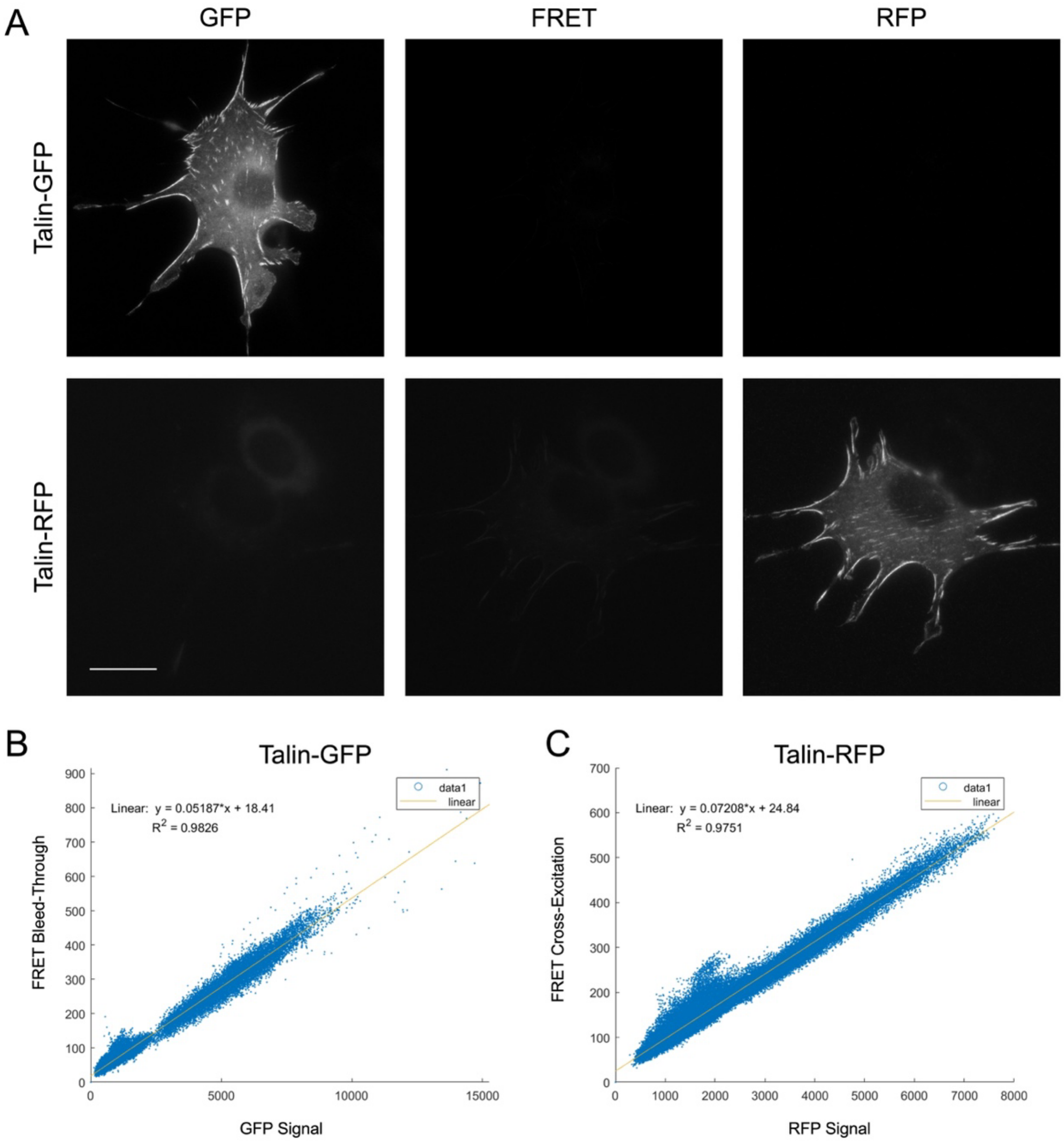

Supplemental Figure 1: Calculation of the bleed through and cross-excitation coefficients for FRET tension sensor imaging using a GFP tagged Talin (Talin-GFP) and an RFP tagged Talin (Talin-RFP). Example images of each construct expressed in 3T3 cells with each of the 3 channels captured for 3-image FRET calculations (A). Plots of GFP bleed through (B) and RFP cross-excitation (C) with linear fits to extract the bleed through and cross-excitation coefficients for the specific microscope settings used. Scale bar = 20μm.

bioRxiv preprint doi: https://doi.org/10.1101/2025.05.09.653153; this version posted May 11, 2025. The copyright holder for this preprint (which was not certified by peer review) is the author/funder, who has granted bioRxiv a license to display the preprint in perpetuity. It is made available under aCC-BY 4.0 International license.

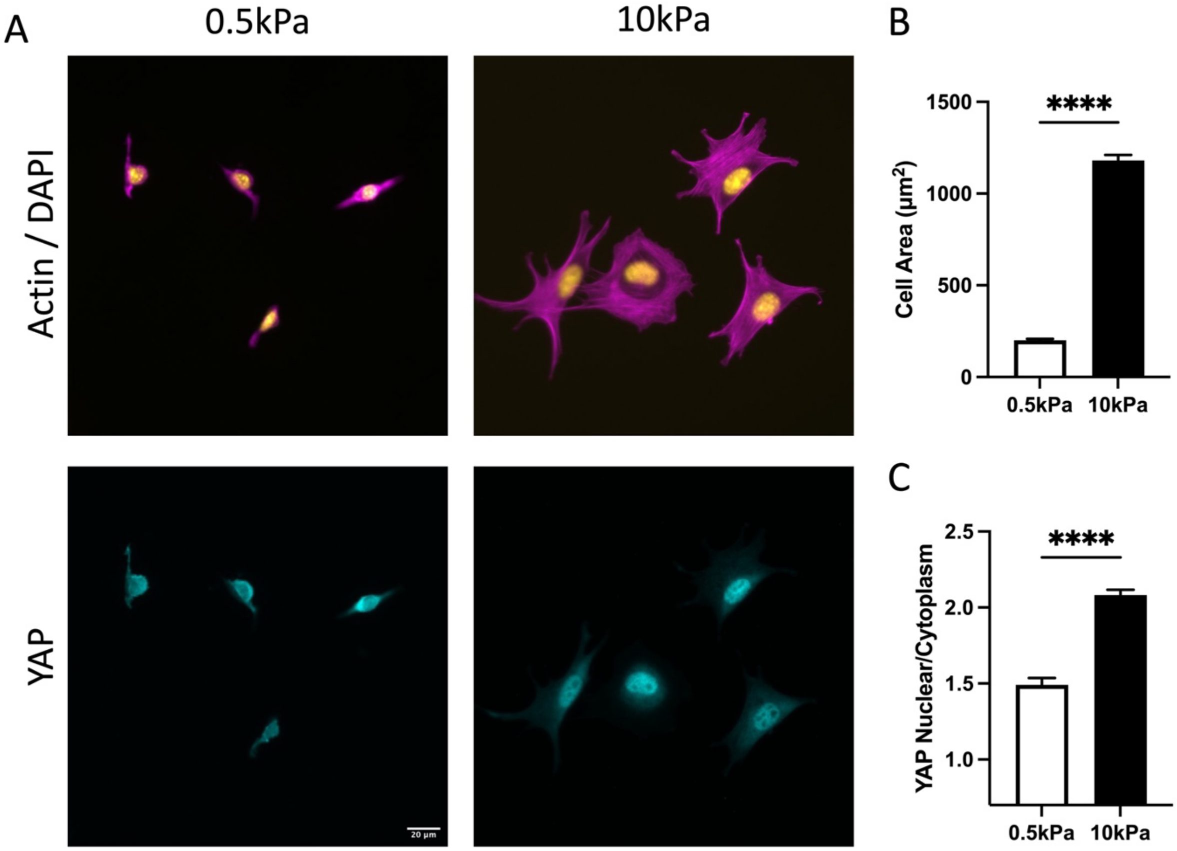

Supplemental Figure 2: Low stiffness control using a 0.5kPA polyacrylamide gel coated with fibronectin and compared to the 10kPA PDMS gel used for TFM and other deformable substrate experiments. (A) Representative images of Actin/DAPI (magenta/yellow) and YAP (cyan). Quantification of cell area (B) and nuclear to cytoplasmic ratio of YAP (C). Mean +/- SEM, two-sided t-test, **** p<0.0001, n=65-190 cells per group. Scale bar = 20μm.

bioRxiv preprint doi: https://doi.org/10.1101/2025.05.09.653153; this version posted May 11, 2025. The copyright holder for this preprint (which was not certified by peer review) is the author/funder, who has granted bioRxiv a license to display the preprint in perpetuity. It is made available under aCC-BY 4.0 International license.

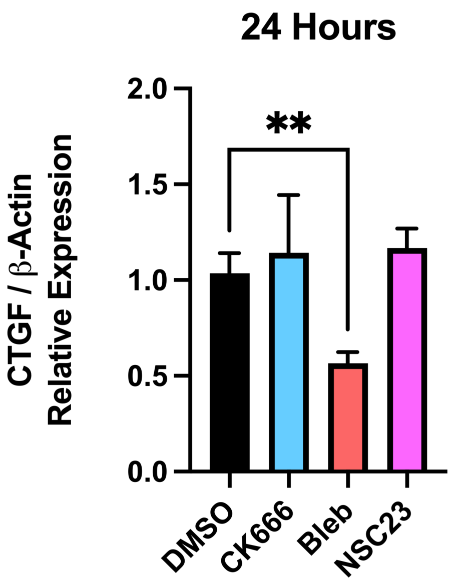

Supplemental Figure 3: qPCR for expression of the YAP target gene CTGF normalized to expression of β-Actin for cells treated with inhibitors for 24 hours on fibronectin coated glass. DMSO (no inhibitor control), inhibition of Arp2/3 (CK666 50μM), inhibition of myosin (Bleb 10 μM) or inhibition of Rac (NSC23 50μM). Mean +/- SEM, n=3-8 samples per group from 3 independent experiments. One-way ANOVA with Bonferroni's Post Hoc, ** $p<0.01$.

bioRxiv preprint doi: https://doi.org/10.1101/2025.05.09.653153; this version posted May 11, 2025. The copyright holder for this preprint (which was not certified by peer review) is the author/funder, who has granted bioRxiv a license to display the preprint in perpetuity. It is made available under aCC-BY 4.0 International license.

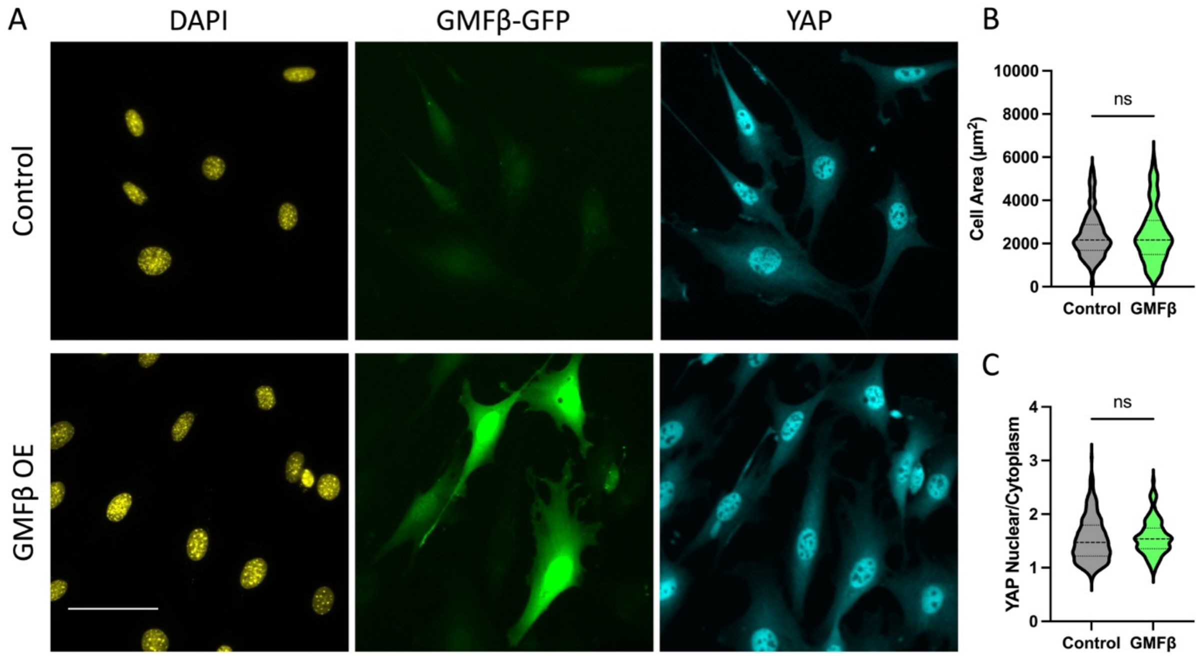

Supplemental Figure 4: Over expression of the actin debranching protein GMFβ-GFP in 3T3 cells seeded for 24 hours on fibronectin coated glass, with representative images (A) of nucleus (DAPI, yellow), GMFβ-GFP (green), and YAP (cyan). Quantification of cell spread area (B) and YAP nuclear to cytoplasmic ratio (C). Violin plots indicate distribution, mean, and quartiles, n>72 cells per group. Scale bar = 50μm.

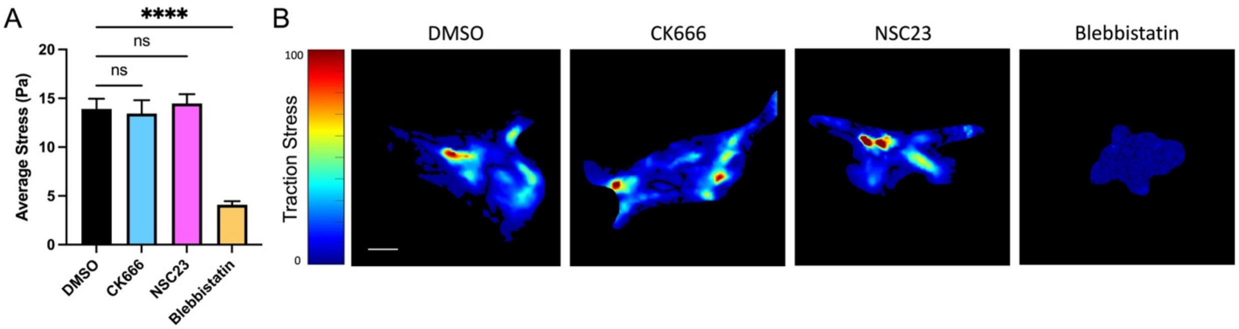

Supplemental Figure 5: Traction force microscopy on 2kPA PDMS gels coated with fibronectin for 3T3 cells treated with Arp2/3 inhibitor (CK666, 50μM), Rac inhibitor (NSC23, 50μM), or myosin inhibitor (Blebbistatin, 10μM), compared to DMSO control. Quantification of average traction stress per cell (A) and representative heat maps of traction forces (B). Mean +/- SEM, n=12-41 cells per group from 2 independent experiments. Scale bar = 10μm.
